# M2 Macrophage-Derived Exosomes Ameliorate BPD by Inhibiting Ferroptosis via Suppression of the ZAKα-p38 Signaling Pathway

**DOI:** 10.3390/antiox15030326

**Published:** 2026-03-05

**Authors:** Yuhan Pu, Mingyue Lv, Ru Yan, Honglian Zhang, Lihui Yu, Weilai Jin, Le Zhang, Zhiwei Yu, Yahui Zhou

**Affiliations:** 1 Department of Pediatrics, Affiliated Children’s Hospital of Jiangnan University (Wuxi Children’s Hospital), Wuxi 210004, China; 2 Department of Neonatology, Affiliated Children’s Hospital of Jiangnan University (Wuxi Children’s Hospital), Wuxi 210004, China; 3 Department of Laboratory, Affiliated Children’s Hospital of Jiangnan University (Wuxi Children’s Hospital), Wuxi 210004, China; 4 Wuxi School of Medicine, Jiangnan University, Wuxi 214122, China

**Keywords:** bronchopulmonary dysplasia (BPD), M2-Exo, ferroptosis, ribosome, ZAKα/p38 pathway

## Abstract

Background: Bronchopulmonary dysplasia (BPD) is a common lung disease in premature infants. Hyperoxia-induced oxidative stress and ferroptosis are key pathological mechanisms leading to alveolar epithelial (AT) cell injury and impaired alveolar development. M2 macrophage-derived exosomes (M2-Exo), as intercellular communication carriers, have potential protective effects in regulating oxidative stress-related diseases, but the molecular mechanism by which they exert effects by regulating ferroptosis in BPD remains unclear. Objective: To explore the protective effect of M2-Exo on hyperoxia or inflammation-induced BPD models and clarify its antioxidant mechanism. Method: In vitro AT cell injury models and in vivo BPD models were constructed by hyperoxia or LPS induction. M2-Exo were isolated, identified, and used to intervene in models. Oxidative stress and ferroptosis-related indicators (ROS, MDA, iron accumulation, GPX4), AT cell functional markers (AQP5, SPC), and ZAKα-p38 pathway activation contents were detected. ZAKα overexpression was used to verify pathway dependence. Results: M2-Exo intervention significantly enhanced AT cell viability, upregulated the expression of AQP5 and SPC, and reversed alveolar simplification. Concurrently, it effectively suppressed hyperoxia or LPS-induced oxidative stress and ferroptosis, as evidenced by reduced contents of ROS and MDA, diminished iron accumulation, and GPX4 expression. Mechanistically, M2-Exo significantly inhibited the activation of the ZAKα-p38 pathway, and ZAKα overexpression could antagonize the antioxidant, anti-ferroptotic, and AT cell protective effects of M2-Exo. Conclusions: M2-Exo alleviate AT cell oxidative stress and ferroptosis by inhibiting the ZAKα-p38 pathway, thereby improving hyperoxia or inflammation-induced BPD and providing a new strategy and molecular target for the antioxidant treatment of BPD.

## 1. Introduction

Bronchopulmonary dysplasia (BPD) remains a major clinical challenge, particularly among premature infants [1]. Although advances in neonatal care have improved survival rates, the incidence and treatment outcomes for BPD have shown limited progress [2].

Thus, identifying effective therapeutic strategies for BPD remains an urgent priority. BPD primarily results from impaired alveolar development, in which alveolar epithelial (AT) cells play a central role. Type 1 alveolar epithelial (AT1) cells cover the alveolar surface and facilitate gas exchange, while type 2 alveolar epithelial (AT2) cells serve as progenitors, capable of self-renewal and differentiation into AT1 cells [3,4].

Recent research has underscored the importance of alveolar macrophages (AMs) in alveolar development. For instance, AM transplantation has been shown to ameliorate pathological features in murine BPD models [5]. Macrophages are broadly categorized into M1 (pro-inflammatory) and M2 (anti-inflammatory) phenotypes [6]. Previous studies have reported that M2 macrophages secrete reparative factors such as Activin A, which contribute to AT2 cell recovery and amelioration of BPD in experimental models [7]. These findings suggest that elucidating the molecular communication between AMs and AT cells could yield novel therapeutic insights for BPD.

Exosomes, key mediators of intercellular communication, have emerged as significant contributors in this context [8]. Macrophages, as secretory cells, release exosomes abundantly. Notably, exosomes derived from M2 macrophages (M2-Exo) have been shown to facilitate bone regeneration and angiogenesis in models of bone defects [9], as well as enhance vascularization and cardiac function following myocardial infarction [10]. These observations raise the possibility that M2-Exo may similarly influence AT cell biology and alveolar development, thereby offering a potential therapeutic avenue in BPD.

In the present study, we demonstrated that M2-Exo alleviated alveolar damage induced by hyperoxia or LPS in established BPD models, both of which are widely employed to mimic BPD-related cellular and tissue damage [11,12]. Damage to AT cells resulting from hyperoxia and inflammation is a critical factor in BPD pathogenesis, involving mechanisms such as oxidative stress and apoptosis [13]. Our data indicate that M2-Exo can attenuate these effects by inhibiting ferroptosis—an iron-dependent form of regulated cell death increasingly implicated in alveolar developmental arrest in BPD. Mechanistically, M2-Exo suppressed ferroptosis by downregulating ZAKα expression and inhibiting p38 MAPK phosphorylation. The ZAKα-p38 signaling pathway, a canonical downstream effector of ribosomal stress, is known to be activated upon ribosomal damage [14]. ZAKα is a core regulator of the ribotoxic stress response (RSR). It senses translation abnormalities through its two C-terminal ribosome-binding domains and triggers the p38 signaling pathway [15]. Zou et al. confirmed that inhibiting p38 phosphorylation can suppress ferroptosis and improve cerebral ischemia, while Huang et al. also verified that inhibiting the p38 signaling pathway inhibits ferroptosis in renal cells [16,17]. These findings suggest that M2-Exo may promote alveolar maturation in BPD through ZAKα-p38 signaling pathway to inhibit ferroptosis.

In summary, this study provides preliminary evidence for the role of M2-Exo in mediating interactions between AMs and AT cells, underscoring their potential in promoting alveolar repair in BPD through mechanisms involving ribosomal stability and suppression of ferroptosis.

## 2. Materials and Methods

### 2.1. Chemicals and Reagents

1640 medium (Gibco, Grand Island, NY, USA, Cat. No. C11875500BT);
Fetal bovine serum (Gibco, Grand Island, NY, USA, USA, Cat. No. A5256701);
Penicillin and streptomycin (Sigma-Aldrich, St. Louis, MO, USA, Cat. No. V900929);
LPS (Sigma-Aldrich, St. Louis, MO, USA, L2630-10MG);
IL-4 (PeproTech, Rocky Hill, NJ, USA, Cat. No. 200-04-20UG);
Exosome isolation kit (Yeasen Biotechnology, Shanghai, China, Cat. No. 41201ES50);
PBS (vivacell, Shanghai, China, Cat. No. C3580-0500);
DIR dye (Thermo Fisher Scientific, Waltham, MA, USA, Cat. No. D12731);
CCK8 (Meilunbio, Dalian, China, Cat. No. MA0218-L);
Apoptosis kit (Vazyme, Nanjing, China, Cat. No. A213-01/02);
ROS assay kit (Beyotime, Shanghai, China, Cat. No. S0033S);
An MDA assay kit (Beyotime, Shanghai, China, S0131S);
FerroOrange assay kit (Dojindo, Kumamoto, Japan, Cat. No. F374);
Trizol (Invitrogen, Carlsbad, CA, USA, Cat. No. 15596026CN);
RIPA lysis buffer (Beyotime, Shanghai, China, Cat. No. P0013B);
5× loading buffer (Epizyme, Shanghai, China, Cat. No. LT101S);
ECL (Vazyme, Nanjing, China, Cat. No. E422);
4% paraformaldehyde (Biosharp, Hefei, China, Cat. No. BL539A);
BSA (Beyotime, Shanghai, China, Cat. No. ST023);
Anti-fading mounting medium containing DAPI (Invitrogen, Carlsbad, CA, USA, Cat. No. P36971);
RNA extraction kit (EZB, Roseville, MN, USA, Cat. No. B0004DP);
Reverse transcription kit: HiScript III RT SuperMix for qPCR (Vazyme, Nanjing, China, Cat. No: R323);
2X ChamQ Universal SYBR qPCR Master Mix (Vazyme, Nanjing, China, Cat. No. Q711);
Polybrene (Sigma-Aldrich, St. Louis, MO, USA, Cat. No. H9268).

### 2.2. Cell Culture

The lung AT II cell line (MLE-12) and alveolar macrophage line (MH-S) obtained from the Chinese Academy of Sciences were cultured in 1640 medium supplemented with 10% fetal bovine serum, 1% penicillin and streptomycin. The cells were incubated in an incubator at 37 °C and 5% CO_2_. Two types of cell models were established to model bronchopulmonary dysplasia (BPD) in vitro. One was induced by hyperoxia, and the other was induced by lipopolysaccharide (LPS). The cells in the hyperoxia group were placed in a high-oxygen incubator and exposed to 85% oxygen. The cells in the LPS group were maintained under normal oxygen conditions (21% O_2_) and treated with 100 ng/mL LPS. In the intervention groups, M2 macrophage-derived exosomes (M2-Exo) were added after hyperoxia or LPS treatment. The final concentration of M2-Exo was 10 µg/mL.

### 2.3. Induced Differentiation of M2 Macrophages

When the alveolar macrophage culture reached 70% confluence, 20 ng/mL IL-4 was added to the medium to stimulate differentiation for 24 h, inducing the polarization of M0 macrophages toward the M2 phenotype. The cells were then continuously cultured for three days, after which the supernatant was collected daily, and a fresh culture medium was added.

### 2.4. Identification of M2 Macrophages

After cell induction was completed according to the aforementioned steps, the phenotype of the macrophages was determined by detecting the surface markers and specific cytokines of M2 macrophages. The markers and cytokines examined included IL-1β, IL-6, Arginase-1 (Arg-1), and CD206.

### 2.5. Isolation and Identification of M2-Exo

The exosomes were extracted using an exosome isolation kit. The specific steps were as follows. Following centrifugation of the collected culture medium at 3000× *g* for 10 min, the supernatant was carefully collected. A volume of 10 mL of the collected cell culture supernatant was transferred to a clean tube, followed by the addition of 2.5 mL of the exosome extraction reagent. The mixture was vortexed for 1 min and incubated at 2–8 °C for 2 h. Subsequently, samples were centrifuged at 10,000× *g* for 60 min. After centrifugation, the supernatant was carefully removed, and the resulting pellet was resuspended in 100 μL of PBS.

### 2.6. Transmission Electron Microscopy (TEM)

Fresh exosome suspensions were adsorbed onto Formvar-coated copper grids placed on Parafilm for 10 min. After removal of excess liquid, grids were fixed with 2.5% glutaraldehyde for 5 min, rinsed with deionized water, and negatively stained with uranyl acetate for 1–10 min at room temperature. Grids were further contrasted with methyl cellulose for 5 min, air-dried or lamp-dried, and examined using a Tecnai transmission electron microscope (FEI, Hillsboro, OR, USA).

### 2.7. Nanoparticle Tracking Analysis (NTA)

Exosome size distribution and particle concentration were characterized using nanoparticle tracking analysis. Particle movement was recorded based on Brownian motion. The sample chamber was rinsed with deionized water followed by PBS to avoid contamination. Exosome samples were diluted 1:500 in PBS before analysis.

### 2.8. Exosomal Labeling (In Vitro Studies)

DIR dye was incubated with M2-Exo at a mass ratio of 1:50 for 1 h to allow labeling. The mixture was subsequently centrifuged at 10,000× *g* for 1 h, and the resulting pellet was resuspended in PBS to a final concentration of 1 μg M2-Exo/μL. DIR-labeled M2-Exo were added to cells at a concentration of 10 μg/μL and incubated for 6 h. Following incubation, the culture medium was removed and cells were washed three times with PBS. While washing, the cells were covered with a prewarmed growth medium and incubated for 10 min, after which the medium was discarded. Fluorescence imaging was performed using a fluorescence microscope (Olympus Corporation, Tokyo, Japan).

### 2.9. Cell Proliferation Assay

Cell proliferation was evaluated by conducting a CCK8 assay. MLE-12 cells were inoculated in 96-well plates at a density of 1 × 10^3^ cells/well and then treated with M2-Exo for 0, 24, 48, 72 h. Absorbance was recorded at 450 nm using a microplate reader (Thermo Fisher, USA).

### 2.10. Cell Apoptosis

The degree of apoptosis was determined using an apoptosis kit. Briefly, 1–5 × 10^5^ cells were collected and centrifuged at 1800 rpm (5 min, 4 °C). The samples were washed twice with precooled PBS. Next, 100 μL of 1× binding buffer was added. The cells were resuspended in a staining solution containing 5 μL of Annexin V-FITC and 5 μL of PI, gently mixed, and incubated in the dark at room temperature for 10 min. Finally, 400 μL of 1× binding buffer was added, and the mixture was gently mixed well. The samples were analyzed by flow cytometry (Becton Dickinson, Franklin Lakes, NJ, USA).

### 2.11. Oxidative Stress

We performed ROS assays using a ROS assay kit. MLE-12 cells were exposed to LPS or hyperoxia for 24 h to induce ROS production; a positive control group was included in the assay. The culture medium was replaced after 24 h of incubation. Rosup solution (1:250), which served as the positive control reagent, was added only to the designated positive control wells and incubated at 37 °C for 30 min. After rinsing once with fresh medium, DCFH-DA was diluted 1:2000 in a serum-free culture medium. The culture medium was removed, and the diluted DCFH-DA solution was added to all the wells except the negative control group. Then, the cells were incubated at 37 °C for 20 min. After incubation, the cells were washed to remove excess dye not taken up by the cells. Finally, the cells were collected, and the ROS contents were analyzed via flow cytometry.

### 2.12. MDA Detection

An MDA assay kit was used to measure MDA contents. Briefly, after 24 h of treatment, 1 × 10^6^ cells were lysed with 0.1 mL of lysis buffer. The lysate was centrifuged at 12,000× *g* for 10 min, and the supernatant was collected. The protein concentration was measured to enable normalization and subsequent calculations. The standard solution was diluted with distilled water to final concentrations of 1, 2, 5, 10, 20, and 50 μM to plot the standard curve. In centrifuge tubes or other suitable containers, 0.1 mL of PBS was used as a blank control, 0.1 mL of each standard solution was added for plotting standard curves, and 0.1 mL of the sample mixture was added for measurement. Subsequently, 0.2 mL of the MDA detection working solution was added to each tube. After thorough mixing, the tubes were heated at 100 °C for 15 min. Samples were cooled to room temperature in a water bath and centrifuged at 1000× *g* for 10 min. Absorbance was recorded at 532 nm using a microplate spectrophotometer. The MDA concentration was calculated following the instructions provided with the kit and normalized to the total protein content. The final MDA contents were expressed as nanomoles per milligram of protein.

### 2.13. Intracellular Fe^2+^ Detection

Cells were plated in fluorescence-compatible culture dishes and incubated overnight at 37 °C in 5% CO_2_. The supernatant was removed, and cells were washed three times with serum-free medium. Cells were then exposed to hyperoxia or stimulated with LPS, with or without M2-Exo treatment. After treatment, cells were washed three times with serum-free medium and incubated with FerroOrange working solution (1 μmol/L) for 30 min at 37 °C in 5% CO_2_. Without additional washing, fluorescence was observed immediately by fluorescence microscopy.

### 2.14. RNA-Seq and Bioinformatics Analysis

Total RNA was isolated from MLE-12 cells using Trizol. The library was prepared following the standard protocol provided by the Shanghai Bohao Institute. Library quality and fragment size distribution were evaluated using an Agilent 4200 Bioanalyzer. High-throughput sequencing was conducted on an Illumina NovaSeq 6000 platform (Illumina, San Diego, CA, USA). Raw sequencing reads were processed using Seqtk (https://github.com/lh3/seqtk, accessed on 13 November 2025) for quality filtering and subsequently mapped to the reference genome with HISAT2 (version: 2.0.4). Differential expression analysis and downstream statistical analyses were performed using the edgeR (version:3.2.0).

### 2.15. Animals

All animal experiments in this study were approved by the Animal Care and Welfare Ethics Committee of Jiangnan University (Approval No. WXCH2022-12-080, dated 20 December 2022) and conducted strictly adhering to relevant national and international guidelines for animal research. Pregnant Sprague–Dawley rats were obtained from the Animal Center of Jiangnan University.

### 2.16. BPD Rat Model

To mimic different pathological factors contributing to BPD, two neonatal Sprague-Dawley rat models were established by exposing them to LPS or hyperoxia. Postnatal day 1 (P1) pups were used for modeling and were housed with their dams under standard conditions (22–25 °C, 50–60% humidity), where the nursing dams had free access to food and water. The experiment was carried out as follows: LPS-induced BPD model: Neonatal rats were randomly assigned to three groups: Con: Rats were raised in room air (21% O_2_) for seven days. LPS: Rats received daily intraperitoneal injections of LPS (500 μg/kg, 50 μL) under ambient air conditions. LPS + M2-Exo: Rats were treated with LPS (500 μg/kg) + M2-Exo (50 μg/kg) in a total volume of 50 μL daily. Hyperoxia-induced BPD model: Neonatal rats were randomly assigned to the following groups: Con: Rats were raised in room air (21% O_2_) for seven days. Hyperoxia (Hyp): Rats were exposed to 85% O_2_ in alternating cycles of 12 h hyperoxia and 12 h normoxia (21% O_2_). Hyp + M2-Exo: Rats received daily intraperitoneal injections of M2-Exo (50 μg/kg, 50 μL) during exposure to hyperoxia. At the end of the experiment, rats were euthanized by cervical dislocation.

### 2.17. In Vivo Biodistribution of M2-Exo

M2-Exo were labeled with the fluorescent dye DIR (the same method used for exosomal labeling). Seven-day-old Sprague–Dawley rats were intraperitoneally injected with DiR-labeled M2-Exo protein, while the control group received intraperitoneal injections of PBS. The lungs, liver, spleen, and small intestine were collected to evaluate fluorescence intensity using a fluorescence imaging instrument (Tanon, Shanghai, China).

### 2.18. Western Blotting

After cell culture, the cells were washed with precooled PBS. RIPA lysis buffer was then added to the cells. Next, the samples were centrifuged at 12,000× *g* (4 °C, 15 min). The supernatant was mixed with 5× loading buffer, vortexed, and heated at 95 °C for 8 min. After electrophoresis, the proteins were transferred onto nitrocellulose membranes. After transfer, the membranes were rinsed with TBST and blocked in 5% skim milk at room temperature for 1.5 h. Excess blocking reagent was removed by washing the membranes with TBST. Primary antibodies were diluted according to the manufacturer’s recommended ratio and incubated with the membranes overnight at 4 °C on a shaker. Details of the primary antibodies used are listed in Table 1. The membranes were washed three times with TBST for 5 min. Secondary antibody dilutions were prepared in TBST following the manufacturer’s instructions. The membranes were incubated with secondary antibodies for 2 h at room temperature. After incubation, the membranes were washed three times in TBST for 5 min each. After TBST washing, the membranes were further rinsed with TBS. Equal volumes of ECL solutions A and B (1:1) were freshly mixed and applied to the membranes. Signals were detected using a chemiluminescence imaging system (Tanon, Shanghai, China), and grayscale analysis was conducted using the ImageJ software (version: 1.53k).

### 2.19. Immunofluorescence

Cells grown on sterile glass coverslips were fixed with 4% paraformaldehyde for 10–20 min, permeabilized with 0.2% Triton X-100 for 5 min, and blocked with 5% BSA at room temperature. Cells were then incubated with primary antibodies overnight, followed by incubation with fluorescent secondary antibodies for 1 h in the dark. After PBST washes, coverslips were mounted with an anti-fade medium containing DAPI. Fluorescence images were acquired using a fluorescence microscope (Olympus, Tokyo, Japan).

### 2.20. Lung Preparation and Histology

On the seventh day, the rats were euthanized, and the lungs were collected. The right lung was fixed in 4% paraformaldehyde for at least 2 h. The lung was then divided into three sections from top to bottom, and 3 μm thick slices were prepared. Lastly, hematoxylin and eosin (H&E) staining was conducted.

### 2.21. RNA Isolation and Quantitative Real-Time PCR (qPCR)

An RNA extraction kit was used to extract RNA. The extracted RNA was placed on ice, and the RNA concentration was measured using an ultramicrospectrophotometer (Thermo Fisher, USA). Next, cDNA was synthesized using a reverse transcription kit. Then, RT-qPCR was performed in mixture using a SYBR Mix. The primer sequences used in this study are listed in Table 2.

### 2.22. Lentiviral Infection

MLE-12 cells were seeded in six-well plates. Viral infection was performed when the cell confluency reached 40–60%. The virus mixture was added to the culture medium at a ratio of 1:10. Polybrene was included during the infection process to increase the infection efficiency. The final concentration of polybrene was 5 μg/mL. After the virus and polybrene were added, the plate was gently mixed. The cells were then placed back into the incubator at 37 °C with 5% CO_2_. The cells were observed 8–12 h post-infection. If no apparent cytotoxicity was detected, the culture medium was left unchanged, and the cells were further incubated for 24 h. The medium was replaced with a fresh culture medium 24 h after infection. For lentiviruses labeled with fluorescence, fluorescence was observed 72–96 h after infection. Infection efficiency was evaluated by determining fluorescence intensity or performing qRT-PCR analysis.

### 2.23. Statistical Analysis

All statistical analyses were conducted using GraphPad Prism 9.0 (GraphPad Software, San Diego, CA, USA). The data are presented as the mean ± SD. Statistical comparisons between two groups were performed using unpaired two-tailed student’s *t*-tests. For comparisons involving more than two groups, one-way ANOVA followed by appropriate post hoc tests was applied. Significance was set at *p* < 0.05.

## 3. Results

### 3.1. Verification of M0 Differentiation into M2 Macrophages Induced by IL-4 and Extraction and Identification of M2-Exo

**Figure 1:**
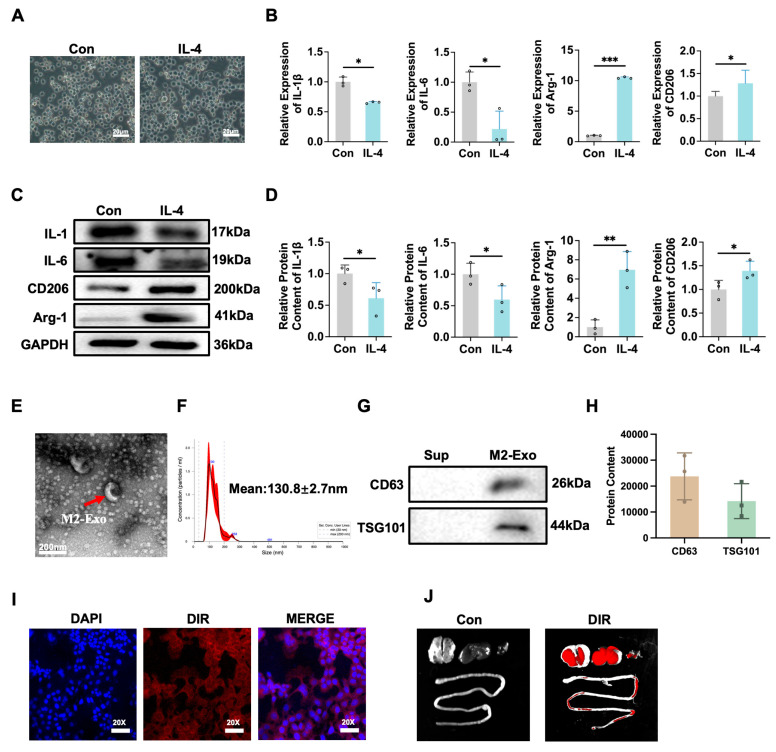
Verification of M0 differentiation into M2 macrophages induced by IL-4 and extraction and identification of M2-Exo. (**A**) Electron microscope images. Scale bars, 20 μm. (**B**) *IL-1β*, *IL-6*, *Arg-1* and *CD206* mRNA relative expression (n = 3). (**C**) Expression of IL-1β, IL-6, Arg-1 and CD206 markers by Western blot (n = 3). (**D**) Quantification of IL-1β, IL-6, Arg-1 and CD206 protein contents normalized with GAPDH contents. (**E**) Representative microscope images of M2-Exo by TEM. Scale bars, 200 nm. (**F**) Nanoparticle tracking analysis (NTA) was used to detect exosome size distribution and concentration. (**G**) Expression of CD63 and TSG101 markers in M2-Exo by Western blot (n = 3). (**H**) Quantification of protein content of CD63 and TSG101. (**I**) The DIR-labeled M2-Exo entered to cells was detected by fluorescence microscope. Original magnification: 20×. (**J**) In vivo imaging of DiR-labeled M2-Exo distribution after injection. Major organs including the lungs, liver, spleen, and small intestine were harvested and subjected to fluorescence imaging using a near-infrared imaging system. A more detailed description of this procedure can be found in the Materials and Methods Section 2.17. Unpaired *t*-test, * *p* < 0.05, ** *p* < 0.01, *** *p* < 0.001. Sup: Supernatant.

To verify the differentiation of M0 macrophages into M2 macrophages, we induced differentiation using IL-4. After treatment, the morphology of the cells exhibited characteristics typical of M2 macrophages (Figure 1A). Quantitative analysis revealed a significant decrease in the expression of inflammatory factors (IL-1β and IL-6) and a significant increase in the expression of anti-inflammatory factors (ARG-1 and CD206) following IL-4 treatment (Figure 1B–D). These findings confirmed the successful differentiation of M0 macrophages into M2 macrophages.

We subsequently extracted M2 macrophage-derived exosomes (M2-Exo) using an exosome extraction kit. Electron microscopy images revealed that M2-Exo exhibited a double-membrane structure, which is characteristic of exosomes (Figure 1E). Nanoparticle tracking analysis revealed that M2-Exo had a diameter of 130.8 ± 2.7 nm, which matched the typical exosome size (Figure 1F). Western blotting analysis confirmed the presence of exosome markers (CD63 and TSG101) in M2-Exo (Figure 1G,H). We also found that DIR-labeled M2-Exo enter cells and that M2-Exo are targeted to lung tissue through intraperitoneal injection (Figure 1I,J). These results validated that the extraction of M2-Exo was successful, providing a foundation for further studies.

### 3.2. Reduction in Hyperoxia-Induced and LPS-Induced Alveolar Epithelial (AT) Cell Damage by M2-Exo May Not Rely on the Inhibition of Apoptosis

**Figure 2:**
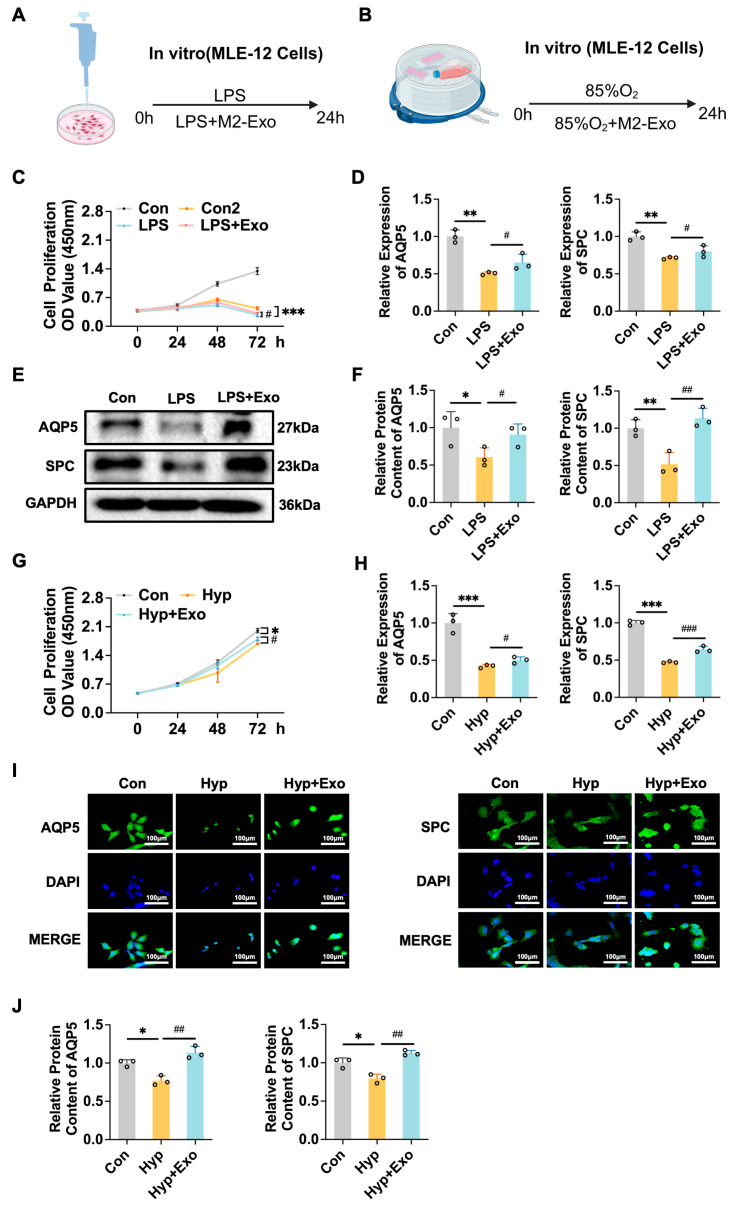
Reduction in hyperoxia-induced and LPS-induced alveolar epithelial (AT) cell damage by M2-Exo may not rely on the inhibition of apoptosis. (**A**,**B**) Schematic illustration of the in vitro BPD cell model induced by LPS or hyperoxia. (**C**,**G**) Proliferation curves of MLE-12 cells co-cultured with M2-Exo (n = 6). Con: Normal control group with 10% FBS, Con2: Normal control group without FBS, LPS: Lipopolysaccharide, Hyp: hyperoxia, Exo: M2-Exo. (**D**,**H**). *AQP5* and *SPC* mRNA relative expression (n = 3). (**E**). Western blots of AQP5 and SPC (n = 3). (**F**) Quantification of AQP5 and SPC protein contents normalized with GAPDH contents. (**I**) Immunofluorescence results, used to evaluate the expression of AQP5 and SPC protein (n = 3). Scale bars, 100 μm. (**J**) Quantification of AQP5 and SPC protein contents. One-way ANOVA, */# *p* < 0.05, **/## *p* < 0.01, ***/### *p* < 0.001.

We assessed the ability of M2-Exo to repair damage in AT cells induced by LPS or hyperoxia (Figure 2A,B). M2-Exo treatment restored the cell viability compromised by LPS or hyperoxia (Figure 2C,G) and improved the mRNA and protein contents of AQP5 and SPC, which are markers of AT 1 and AT 2 cells, respectively (Figure 2D–F,H–J). These results suggest that M2-Exo can facilitate the repair of AT cell damage caused by LPS or hyperoxia.

Apoptosis plays a crucial role in AT cell damage. Our previous studies indicated that inhibiting apoptosis can ameliorate AT cell damage in BPD models. In this study, M2-Exo significantly reduced hyperoxia-induced apoptosis but did not affect LPS-induced apoptosis (Figure S1A,B). Analysis of the apoptosis-related markers BCL-2 (antiapoptotic) and BAX (proapoptotic) revealed that M2-Exo improved the expression of *BCL-2* mRNA and reduced the expression of *BAX* mRNA in the hyperoxia and LPS models but did not affect protein contents (Figure S1C–H). To further investigate apoptotic signaling, we evaluated cleaved-caspase3 expression (Figure S1I–L). Hyperoxia/LPS increased cleaved-caspase3 contents compared with the control group. However, M2-Exo treatment did not mitigate this increase. M2-Exo had minimal impact on cleaved-caspase3 contents in this context.

### 3.3. M2-Exo Can Inhibit Ferroptosis Induced by LPS or Hyperoxia

**Figure 3:**
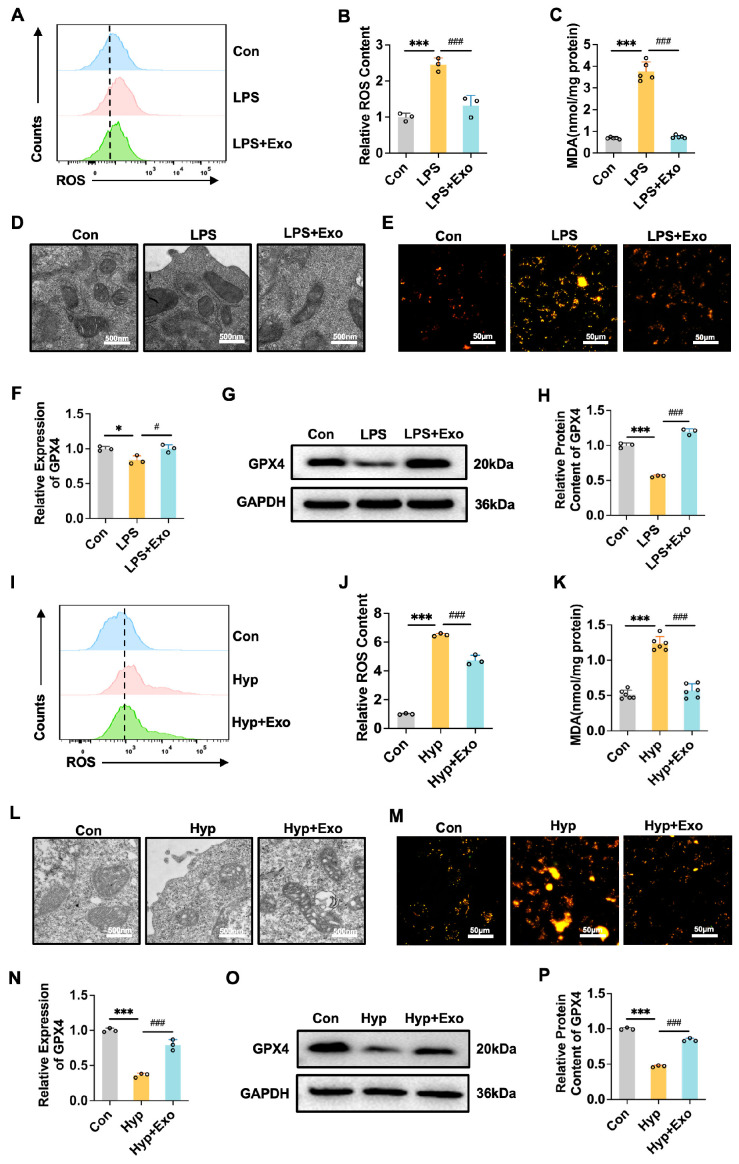
M2-Exo inhibits ferroptosis induced by LPS or hyperoxia exposure. (**A**,**I**) Cellular oxidative stress (ROS) was detected by flow cytometry (n = 3). (**B**,**J**) Quantification of ROS contents. (**C**,**K**) MDA content of cells tested by MDA kit (n = 6). (**D**,**L**) Mitochondrial morphology of MLE-12 cells under transmission electron microscope. Scale bars, 500 nm. (**E**,**M**) Intracellular Fe^2+^ levels detected by a fluorescent probe. Scale bars, 50 μm. (**F**,**N**) *GPX4* mRNA relative expression (n = 3). (**G**,**O**) Western blots of GPX4 (n = 3). (**H**,**P**) Quantification of GPX4 protein contents normalized with GAPDH contents. One-way ANOVA, */# *p* < 0.05, ***/### *p* < 0.001.

Oxidative stress is a key factor in AT cell injury. Our results revealed that M2-Exo treatment reduced the contents of ROS induced by LPS or hyperoxia (Figure 3A,B,I,J) and decreased the expression of MDA (Figure 3C,K). Morphological analysis revealed that M2-Exo alleviated iron accumulation in mitochondria caused by LPS or hyperoxia. These iron deposits were observed as electron-dense granules within the mitochondrial matrix, often accompanied by outer membrane rupture and loss of cristae structure (Figure 3D,L). To further confirm whether iron accumulation occurred in these cells, we employed a specific fluorescent probe for divalent ferrous iron (Fe^2+^) to assess intracellular iron contents across different experimental groups (Figure 3E,M). The results showed that both LPS and hyperoxia treatments led to substantial iron accumulation, while M2-Exo treatment markedly reduced Fe^2+^ accumulation. Additionally, M2-Exo increased the mRNA and protein contents of GPX4, which were downregulated by LPS and hyperoxia (Figure 3F–H,N–P). These results indicated that M2-Exo may inhibit ferroptosis, thereby protecting AT cells from damage induced by LPS or hyperoxia.

### 3.4. M2-Exo Can Inhibit Ferroptosis In Vivo to Improve the BPD Model Induced by LPS or Hyperoxia

**Figure 4:**
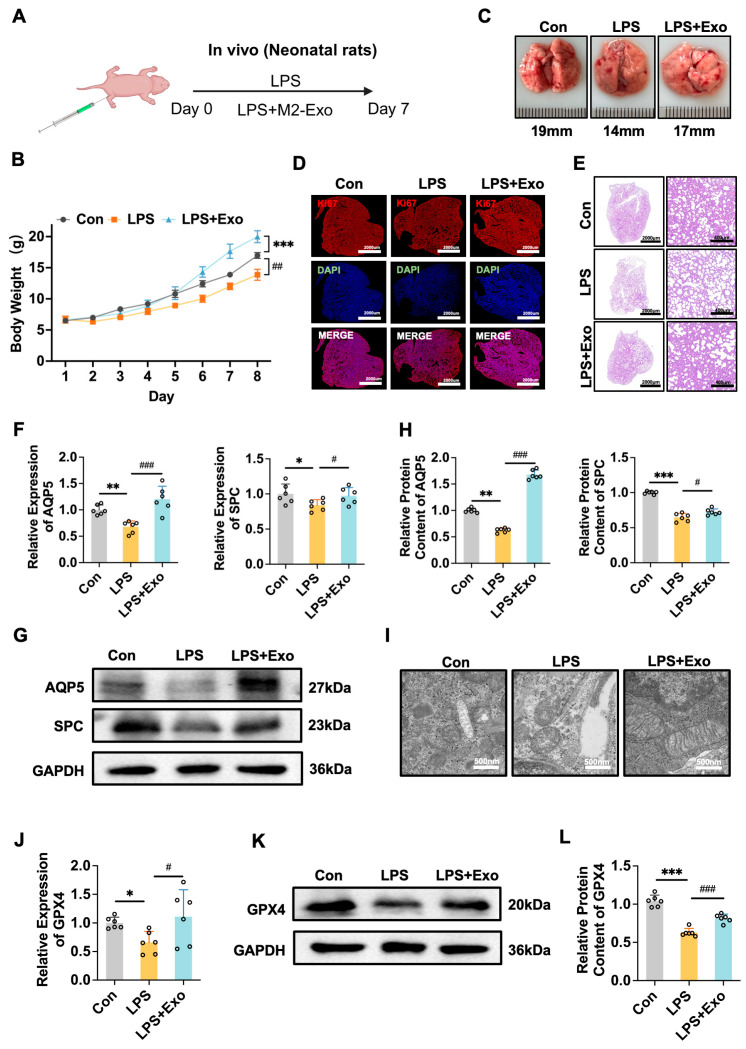
M2-Exo can inhibit ferroptosis in vivo to improve the BPD model induced by LPS. (**A**) Schematic illustration of the in vivo BPD animal model induced by LPS. (**B**) The body weight change (n = 6). (**C**) The morphology of lung tissue. (**D**) Ki67 immunostaining was performed to evaluate lung tissue proliferation. Scale bars, 2000 μm. (**E**) Histopathological changes in the lung by H&E. Scale bars, 2000/400 μm. (**F**) *AQP5* and *SPC* mRNA relative expression (n = 6). (**G**) Western blots of AQP5 and SPC (n = 6). (**H**) Quantification of AQP5 and SPC protein contents normalized with GAPDH contents. (**I**) Under LPS conditions, the iron accumulation in mitochondria of AT cells observed by transmission electron microscopy. Scale bars, 500 nm. (**J**) *GPX4* mRNA relative expression (n = 6). (**K**) Western blots of GPX4 (n = 6). (**L**) Quantification of GPX4 protein contents normalized with GAPDH contents. One-way ANOVA, */# *p* < 0.05, **/## *p* < 0.01, ***/### *p* < 0.001.

**Figure 5:**
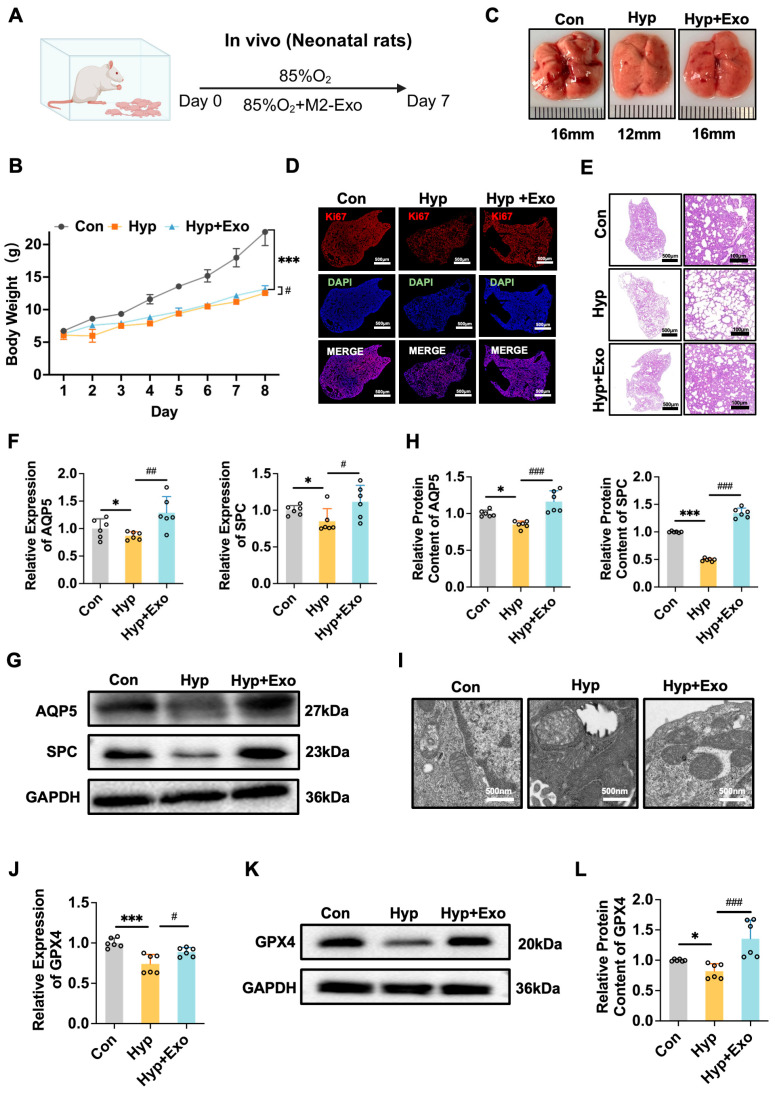
M2-Exo can inhibit ferroptosis in vivo to improve the BPD model induced by hyperoxia. (**A**) Schematic illustration of the in vivo BPD animal model induced by hyperoxia. (**B**) The body weight change (n = 6). (**C**) The morphology of lung tissue. (**D**) Ki67 immunostaining was performed to evaluate lung tissue proliferation. Scale bars, 500 μm. (**E**) Histopathological changes in the lung by H&E. Scale bars, 500/100 μm. (**F**) *AQP5* and *SPC* mRNA relative expression (n = 6). (**G**) Western blots of AQP5 and SPC (n = 6). (**H**) Quantification of AQP5 and SPC protein contents normalized with GAPDH contents. (**I**) The iron ions accumulate in mitochondria of type II alveolar epithelial cells observed by transmission electron microscopy. Scale bars, 500 nm. (**J**) *GPX4* mRNA relative expression (n = 6). (**K**) Western blots of GPX4 (n = 6). (**L**) Quantification of GPX4 protein contents normalized with GAPDH contents. One-way ANOVA, */#*p* < 0.05, ##*p* < 0.01, ***/###*p* < 0.001.

To evaluate the effectiveness of M2-Exo in vivo, we administered M2-Exo to BPD model pups via intraperitoneal injection (Figure 4A and Figure 5A). M2-Exo treatment significantly mitigated the weight loss caused by LPS or hyperoxia (Figure 4B and Figure 5B) and improved the lung tissue structure (Figure 4C and Figure 5C). H&E staining revealed that M2-Exo improved the pathological features of BPD, including alveolar simplification, reduced alveolar number, and widened alveolar intervals (Figure 4E and Figure 5E). M2-Exo also increased the expression of Ki67, a marker of cell proliferation (Figure 4D and Figure 5D). Additionally, M2-Exo restored AT cell marker expression altered by LPS or hyperoxia (Figure 4F–H and Figure 5F–H). Electron microscopy images revealed a decrease in iron accumulation in lung mitochondria following treatment with M2-Exo (Figure 4I and Figure 5I). Additionally, M2-Exo increased GPX4 expression in BPD models (Figure 4J–L and Figure 5J–L). These results suggest that M2-Exo can inhibit ferroptosis and enhance alveolar development, thereby improving the BPD phenotype.

### 3.5. M2-Exo Can Inhibit Ferroptosis Induced by Ferroptosis Activators

**Figure 6:**
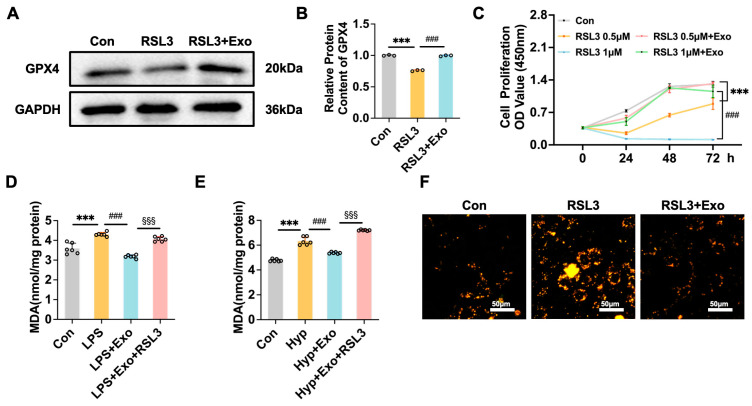
M2-Exo can inhibit ferroptosis induced by ferroptosis activators. (**A**) Western blots of GPX4 (n = 3). (**B**) Quantification of GPX4 protein contents normalized with GAPDH contents. (**C**) Proliferation curves of MLE-12 cells (n = 6). (**D**,**E**) MDA content of cells tested by MDA kit (n = 6). (**F**) Intracellular Fe^2+^ levels detected by a fluorescent probe. Scale bars, 50 μm. One-way ANOVA, ***/###/§§§ *p* < 0.001.

To determine whether M2-Exo can inhibit ferroptosis, a ferroptosis activator (RSL3) was added to induce ferroptosis. RSL3 significantly inhibited the expression of GPX4, whereas M2-Exo intervention reversed this effect of RSL3 (Figure 6A,B). Similarly, M2-Exo significantly improved the decrease in cell viability induced by RSL3 (Figure 6C). Moreover, RSL3 inhibited the effect of M2-Exo on the increase in MDA contents induced by hyperoxia or LPS (Figure 6D,E). M2-Exo also attenuated iron accumulation induced by the ferroptosis activator RSL3 (Figure 6F). These results suggest that M2-Exo can inhibit ferroptosis to improve cell conditions.

### 3.6. Determining the Mechanism by Which M2-Exo Inhibit Ferroptosis to Repair AT Cell Damage and Improve Alveolar Development in BPD

**Figure 7:**
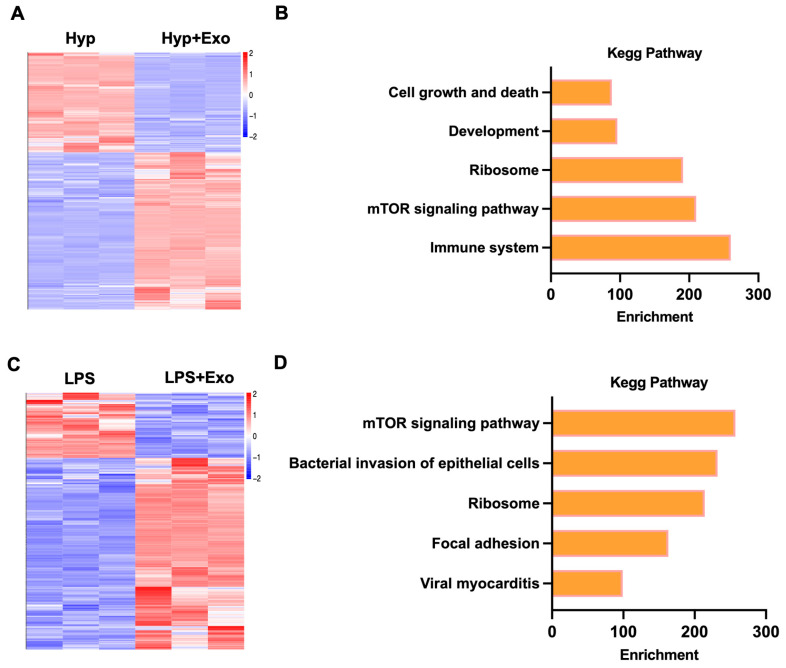
Determining the mechanism by which M2-Exo inhibit ferroptosis to repair AT cell damage and improve alveolar development in BPD. (**A**,**C**) Heatmap analysis of the DEGs. (**B**,**D**) KEGG pathway analysis of DEGs.

RNA sequence analysis was performed to elucidate the mechanisms through which M2-Exo inhibit ferroptosis. A total of 1302 differentially expressed genes (DEGs) were identified between hyperoxia and hyperoxia with M2-Exo treatment. Among these genes, 820 were upregulated and 482 were downregulated in the M2-Exo group compared to the hyperoxia group (log2-fold change ≤ −2 or ≥2, *p* < 0.05) (Figure 7A). In the LPS-treated and LPS-treated M2-Exo groups, 117 DEGs were identified, with 85 upregulated genes and 32 downregulated genes in the M2-Exo group (log2-fold change ≤ −2 or ≥2, *p* < 0.05) (Figure 7C). The results of the KEGG pathway analysis revealed that these DEGs were involved primarily in the ribosome and mTOR signaling pathways (Figure 7B,D). These findings indicated that M2-Exo may inhibit ferroptosis and repair AT cells by modulating ribosome and mTOR signaling pathways.

### 3.7. M2-Exo Can Inhibit the Downstream Effects of the Ribosome ZAKα-p38 Pathway

**Figure 8:**
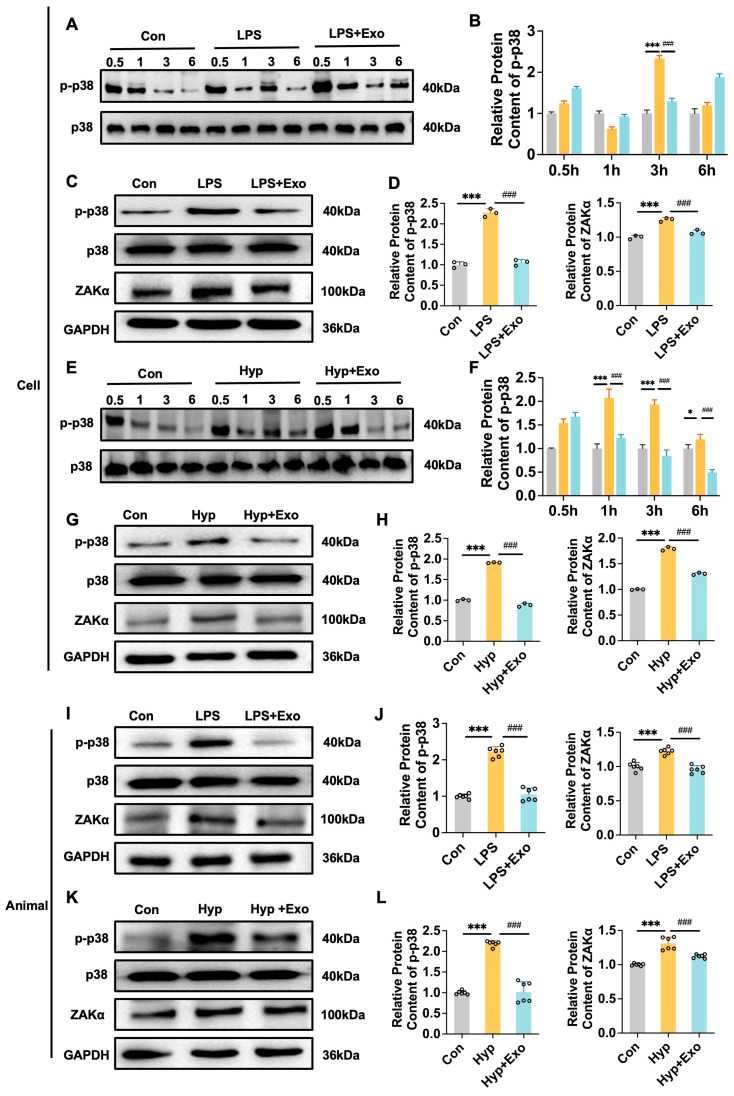
M2-Exo can inhibit the downstream effects of the ribosome ZAKα-p38 pathway. (**A**,**E**) Western blots of p-p38 at different time points (n = 3). (**B**,**F**) Quantification of p-p38 protein contents normalized with p38 contents. (**C**,**G**) Western blots of p-p38 and ZAKα in cells (n = 3). (**D**,**H**) Quantification of p-p38 and ZAKα protein contents normalized with p38/GAPDH contents. (**I**,**K**) Western blots of p-p38 and ZAKα in rats (n = 6). (**J**,**L**) Quantification of p-p38 and ZAKα protein contents normalized with GAPDH contents. One-way ANOVA, * *p* < 0.05, ***/### *p* < 0.001.

Owing to the significant enrichment of the mTOR signaling pathway, which can activate ferroptosis, we aimed to determine whether M2-Exo can improve BPD by inhibiting this pathway. Activation of the mTOR signaling pathway primarily promotes AKT phosphorylation. However, our results revealed that M2-Exo did not alter AKT phosphorylation contents at multiple time points (Figure S2A–D). Additionally, srebp1 and scd1, upstream regulators of ferroptosis activation, can be activated by the mTOR signaling pathway. However, our findings indicated that in the LPS model, srebp1 and scd1 were activated, and M2-Exo inhibited LPS-induced activation. In contrast, in the hyperoxia model, srebp1 and scd1 exhibited a trend opposite to that found in the LPS model (Figure S2E,F). These results suggest that the mTOR signaling pathway may not be the primary mechanism through which M2-Exo inhibits ferroptosis to improve BPD. We then investigated the ribosome-related pathways. Previous studies have identified ZAKα as a downstream regulator of ribosomes. Our results revealed that the exposure of cells to LPS or hyperoxia increased ZAKα expression and p38 phosphorylation, whereas M2-Exo treatment reversed these effects (Figure 8A–H). These findings were similar to those reported in animal models (Figure 8I–L), which suggested that M2-Exo may stabilize ribosomes, inhibit ZAKα expression, and prevent p38 phosphorylation, thereby reducing ferroptosis and repairing damaged AT cells.

### 3.8. Overexpression of ZAKα Weakened the Ability of M2-Exo to Inhibit Ferroptosis and Induce AT Repair in Cells

**Figure 9:**
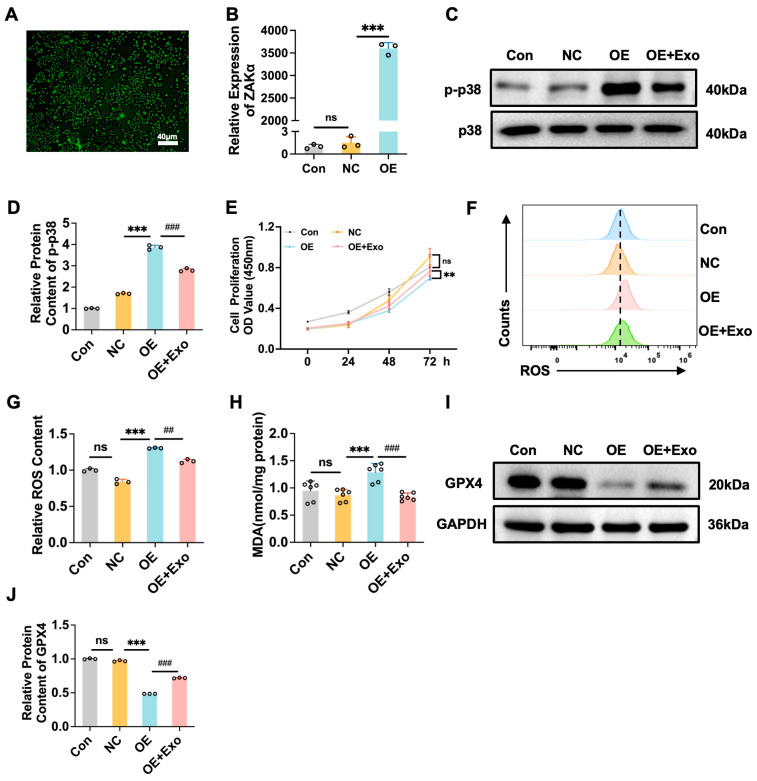
Overexpression of ZAKα weakened the ability of M2-Exo to inhibit ferroptosis and induce AT repair in cells. (**A**) Overexpression of ZAKα fluorescence intensity. Scale bars, 40 μm. (**B**) ZAKα overexpression efficiency detected by PCR. (**C**) Western blots of p-p38 (n = 3). (**D**) Quantification of p-p38 protein contents normalized with p38 contents. (**E**) Proliferation curves of MLE-12 cells (n = 6). (**F**) Cellular oxidative stress (ROS) was detected by flow cytometry (n = 3). (**G**) Quantification of ROS contents. (**H**) MDA content of cells tested by MDA kit (n = 6). (**I**) Western blots of GPX4 (n = 3). (**J**) Quantification of GPX4 protein contents normalized with GAPDH contents. NC: Negative Control, OE: overexpression of ZAKα. One-way ANOVA, ns *p* > 0.05, **/## *p* < 0.01, ***/### *p* < 0.001.

To assess the role of ZAKα in the therapeutic effect of M2-Exo, we significantly overexpressed ZAKα in AT cells (Figure 9A,B). Overexpressing ZAKα significantly inhibited the phosphorylation of p38, whereas M2-Exo significantly increased the phosphorylation of p38 induced by the overexpression of ZAKα (Figure 9C,D). The overexpression of ZAKα substantially decreased cell viability. However, M2-Exo significantly improved the decrease in cell viability induced by ZAKα overexpression (Figure 9E). Similarly, M2-Exo also significantly alleviated the increase in ROS and MDA contents induced by ZAKα overexpression (Figure 9F–H). By assessing the classical ferroptosis marker GPX4, we found that M2-Exo significantly reversed the inhibitory effect of ZAKα overexpression on the expression of GPX4 (Figure 9I,J). These results indicated that M2-Exo may inhibit ferroptosis and repair AT cell damage by stabilizing ribosomes and activating ZAKα.

## 4. Discussion

Bronchopulmonary dysplasia (BPD) is a significant challenge in neonatology because of its high incidence and limited treatment options. The pathogenesis of BPD involves complex factors, including prenatal and postnatal inflammation, as well as impaired alveolar development due to prolonged exposure to hyperoxia [18,19]. AM and AT cells are the main constituent cells of the alveoli, and they are often damaged by these factors. Although AM and AT cells are independent, they also interact. A study published in 2024 reported that the interaction between lung AT cells and innate immune cells can more effectively repair acute lung injury and maintain lung homeostasis; it also stated that AM cells are the main immune cells in the lungs that are in homeostasis, mainly to inhibit inflammation [20]. They communicate with each other via different mechanisms, including mediators, membrane glycoproteins, extracellular vesicles, and gap junction channels [21]. Another study reported that renal tubular epithelial cells and macrophages form a negative feedback loop through exosome transfer, which promotes renal inflammation and apoptosis in diabetic nephropathy [22]. Renal macrophages and collecting duct epithelial cells interact through exosomes to drive tubulointerstitial inflammation and fibrosis in lgA nephropathy [23]. Although no reports have yet described the role of exosomes in mediating interactions between AM and AT cells in the development of BPD, their function as a crucial communication medium suggests that further investigation may offer a novel strategy for the prevention and treatment of BPD.

A study reported that M1 macrophages secrete mainly proinflammatory factors, which can activate lung inflammation and induce the progression of BPD [24]. M2 macrophages normally secrete inhibitory factors that contribute to cell proliferation and tissue recovery [25] and repair BPD-like alveolar damage [26,27]. In this study, we induced M2 macrophages using IL-4 and subsequently extracted M2-Exo to assess their role in influencing AT cells. Our findings demonstrated that M2-Exo significantly enhanced the proliferation of AT cells and mitigated damage induced by hyperoxia or LPS. These results suggested that M2-Exo may alleviate AT injury in models of BPD.

Apoptosis contributes to alveolar damage in BPD, and inhibiting apoptosis promotes alveolar development and reduces the progression of BPD [13,28]. Our study revealed that M2-Exo partially inhibited the apoptosis induced by LPS or hyperoxia; however, the effect was not consistent across all conditions. This variability suggests additional mechanisms by which M2-Exo may exert protective effects in BPD models. Recent studies have implicated ferroptosis, a form of regulated cell death driven by iron-dependent lipid peroxidation, in the pathogenesis of BPD. Ming Yang et al. demonstrated that ETSI inhibits ferroptosis to improve alveolar development in a BPD mouse model [29], while Yifan Luo et al. reported that many ferroptosis-related genes participate in BPD pathogenesis [30]. Our study revealed that M2-Exo effectively inhibited ferroptosis induced by LPS and hyperoxia. Ferroptosis activation counteracted the protective effects of M2-Exo, highlighting the importance of ferroptosis inhibition in mediating the reparative effects of M2-Exo on AT cells.

This study adopted a multi-model construction strategy to carry out research on the mechanisms related to BPD. On the one hand, hyperoxia exposure was selected to establish the classical BPD model since it can accurately simulate the core pathogenic scenario of hyperoxia exposure in preterm neonates in clinical practice, and its pathological processes are highly consistent with those of human BPD [31]; on the other hand, it was selected because postnatal sepsis is being increasingly recognized as a major risk factor for the occurrence of BPD, and recent studies have confirmed that sustained LPS exposure exerts its effects by regulating the expression of chemokine CCL2, which has been proven to be a key molecule mediating BPD induced by hyperoxia exposure [12]. Therefore, we simultaneously established an inflammation-related BPD model via LPS exposure according to the method described by Amrit Kumar Shrestha et al. [32]. In addition, this study intended to explore the core mechanism by which M2 macrophage-derived exosomes improve BPD by repairing alveolar epithelial cell damage; the application of the LPS modeling method can more specifically confirm the potential mechanism by which M1 macrophages promote the occurrence of BPD by driving inflammatory responses, thereby providing more sufficient experimental evidence for comprehensively clarifying the regulatory role of macro-phages in BPD and defining the therapeutic value of M2 macrophage-derived exosomes.

However, the mechanism by which M2-Exo inhibit ferroptosis is not clear. To elucidate the mechanisms underlying ferroptosis inhibition by M2-Exo, we conducted RNA sequencing (RNA-seq) analysis, which revealed differential gene expression primarily involving the mTOR and ribosome pathways. While modulation of the mTOR pathway has been linked to ferroptosis regulation in various contexts—for example, SCD1 can inhibit ferroptosis through the SQLE/cholesterol/mTOR signaling pathway to promote the stemness of gastric cancer stem cells [33]—knocking down TYMS promotes ferroptosis via inhibition of the PI3K/Akt/mTOR signaling pathway to suppress cell proliferation in triple-negative breast cancer [34]. BCAT1 inhibits ferroptosis through the PI3K/AKT/mTOR/GPX4 pathway and alleviates early brain injury [35]. We did not find significant involvement of this pathway in the M2-Exo-mediated effects on ferroptosis. M2-Exo may inhibit ferroptosis in other ways.

Our results revealed that DEGs are involved in ribosomes. Another study reported that ribosome damage may injure cells by depleting the number of productive ribosomes and producing truncated or abnormal proteins through frameshifts [36]. The ZAKα-p38 pathway is the downstream pathway of the ribosome. ROS can trigger ribosome damage to activate the ZAKα-p38 signaling pathway [37]. Additionally, ribosomal stress-induced ZAKα/p38 activation promotes the release of the NLRP1 inflammasome. We identified ribosomal pathways and downstream ZAKα-p38 signaling as potential mediators of the ferroptosis-inhibiting effects of M2-Exo [14]. Our results revealed that the DEGs between the LPS-treated and LPS + M2-Exo groups or between the hyperoxia-treated and hyperoxia + M2-Exo groups involved ribosomes. Hyperoxia or LPS induced the expression of ROS and increased ZAKα/p38 activation, but M2-Exo not only inhibited ROS but also inhibited ZAKα expression and p38 phosphorylation. This finding matches previously reported results, indicating that an increase in ROS contents blocks stable ribosome activation of the ZAKα/P38 pathway and subsequently damages cells [37]. M2-Exo appeared to stabilize ribosomes and suppress the activation of ZAKα-p38, thereby reducing ferroptosis and promoting AT cell repair. Additionally, the overexpression of ZAKα reversed the protective effects of M2-Exo on AT cells, highlighting that the ZAKα-p38 pathway plays an important role in mediating the therapeutic efficacy of M2-Exo in BPD models. These findings suggest a novel mechanism whereby M2-Exo may stabilize ribosomes to inhibit ferroptosis and protect against alveolar damage in BPD.

## 5. Conclusions

In conclusion, our study provides preliminary evidence that M2-Exo can effectively inhibit ferroptosis and repair AT cell damage in models of BPD induced by hyperoxia and LPS. The underlying mechanism involves stabilizing ribosomes to suppress the ZAKα-p38 pathway, thereby mitigating ferroptosis and promoting alveolar development. This research not only sheds light on the interactions between AM and AT cells via exosomes but also suggests new avenues for the treatment and prevention of BPD. Future studies will aim to further elucidate the mechanisms by which M2-Exo stabilize ribosomes and identify specific bioactive components within M2-Exo, addressing current study limitations.

## Figures and Tables

**Table 1:** The information of antibodies in this study.

Antibody	Source	Supplier	Catalog Number	Dilution
GAPDH	Mouse	Proteintech	Cat#60004-1-Ig	1:50,000
IL-1β	Rabbit	Novusbio	Cat#NB600-633	1:1000
IL-6	Rabbit	Novusbio	Cat#NB600-1131SS	1:1000
CD206	Rabbit	CST	Cat#24595	1:1000
Arg-1	Rabbit	Proteintech	Cat#16001-1-AP	1:5000
TSG101	Rabbit	Proteintech	Cat#28283-1-AP	1:2000
CD63	Rabbit	Proteintech	Cat#25682-1-AP	1:1000
SFTPC	Rabbit	Proteintech	Cat#10774-1-AP	1:1000
AQP5	Rabbit	Proteintech	Cat#20334-1-AP	1:1000
GPX4	Rabbit	Proteintech	Cat#67763-1-Ig	1:1000
p-P38	Rabbit	Proteintech	Cat#28796-1-AP	1:1000
ZAKα	Rabbit	Abclonal	Cat#A7371	1:1000
BCL-2	Mouse	Proteintech	Cat#68103-1-Ig	1:5000
BAX	Rabbit	Proteintech	Cat#50599-2-Ig	1:2000
Cleaved-caspase3	Mouse	Proteintech	Cat#68773-1-Ig	1:2000
p-AKT(Ser473)	Mouse	Proteintech	Cat#66444-1-Ig	1:2000

**Table 2:** The sequence of the primer.

Name	Sequence	Gene Numbers
Mouse *GAPDH*-F	TGGATTTGGACGCATTGGTC	NM_008085.2
Mouse *GAPDH*-R	TTTGCACTGGTACGTGTTGAT	
Mouse *IL-1β*-F	GCAACTGTTCCTGAACTCAACT	NM_008361.4
Mouse *IL-1β*-R	ATCTTTTGG GGTCCGTCAACT	
Mouse *IL-6*-F	TAGTCCTTCCTACCCCAATTTCC	NM_031168.2
Mouse *IL-6*-R	TTGGTCCTTAGCCACTCCTTC	
Mouse *Arg-1*-F	CTCCAAGCCAAAGTCCTTAGAG	NM_007482.3
Mouse *Arg-1*-R	GGAGCTGTCATTAGGGACATCA	
Mouse *CD206*-F	CTCTGTTCAGCTATTGGACGC	NM_008625.2
Mouse *CD206*-R	CGGAATTTCTGGGATTCAGCTTC	
Mouse *AQP5*-F	AGAAGGAGGTGTGTTCAGTTGC	NM_009701.4
Mouse *AQP5*-R	GCCAGAGTAATGGCCGGAT	
Mouse *SPC*-F	ATGGACATGAGTAGCAAAGAGGT	NM_011359.2
Mouse *SPC*-R	CACGATGAGAAGGCGTTTGAG	
Mouse *GPX4*-F	GATGGAGCCCATTCCTGAACC	NM_001037741.4
Mouse *GPX4*-R	CCCTGTACTTATCCAGGCAGA	
Mouse *Bax*-F	AGCCACAAAGATGGTCACT	NM_001411996.1
Mouse *Bax*-R	GGAGATGAACTGGATAGCAA	
Mouse *Bcl-2*-F	ATCTCCCTGTTGACGCTCT	NM_177410.3
Mouse *Bcl-2*-R	CATCTTCTCCTTCCAGCCT	
Mouse *ZAKα*-F	AACTATGGCATCGTCACAGA	NM_023057.6
Mouse *ZAKα*-R	TGTGTGTTGTGTGGTTATGGA	
Mouse *Srebf1*-F	AACGTCACTTCCAGCTAGAC	NM_001358315.1
Mouse *Srebf1*-R	CCACTAAGGTGCCTACAGAGC	
Mouse *Scd1*-F	TCCAACTCATGTGCCTCTGT	NM_009127.4
Mouse *Scd1*-R	AACAACCAACCCTCGCATTC	
Rat *GAPDH*-F	CGGAGTCAACGGATTTGGTCGTAT	NM_017008.4
Rat *GAPDH*-R	AGCCTTCTCCATGGTGGTGAAGAC	
Rat *SPC*-F	CTGAGATGGTCCTTGAGATGAG	NM_017342.2
Rat *SPC*-R	AATAGAGAAGGTAGCGATGGTG	
Rat *AQP5*-F	GAGATTCGTGAATGCGGTGC	NM_012779.2
Rat *AQP5*-R	AATGTCCCCTCTGTCCACCT	
Rat *GPX4*-F	AGCAAGATCTGTGTAAATGGG	NM_017165.4
Rat *GPX4*-R	TTTGATGGCATTTCCCAGC	

## Data Availability

The data that support the findings of this study are available from the corresponding author, Yahui Zhou. The RNA sequencing data presented in the study are openly available in the Gene Expression Omnibus (GEO) da-tabase under accession number GSE322753.

## Supplementary Materials

The following supporting information can be downloaded at: https://www.mdpi.com/article/10.3390/antiox15030326/s1, Figure S1: The role of M2-Exo inhibits apoptosis in LPS or hyperoxia induced BPD cell model; Figure S2: M2-Exo intervention has no role in mTOR signalling pathway.
