# Supplementary material for: M2 Macrophage-Derived Exosomes Ameliorate BPD by Inhibiting Ferroptosis via Suppression of the ZAKα-p38 Signaling Pathway
Source: Antioxidants (Basel). 2026 Mar 5;15(3):326. doi: 10.3390/antiox15030326 (PMC13023919; doi:10.3390/antiox15030326)
Supplement: Supplementary file 1 [file antioxidants-15-00326-s001.zip › antioxidants-4015741-supplementary.pdf]

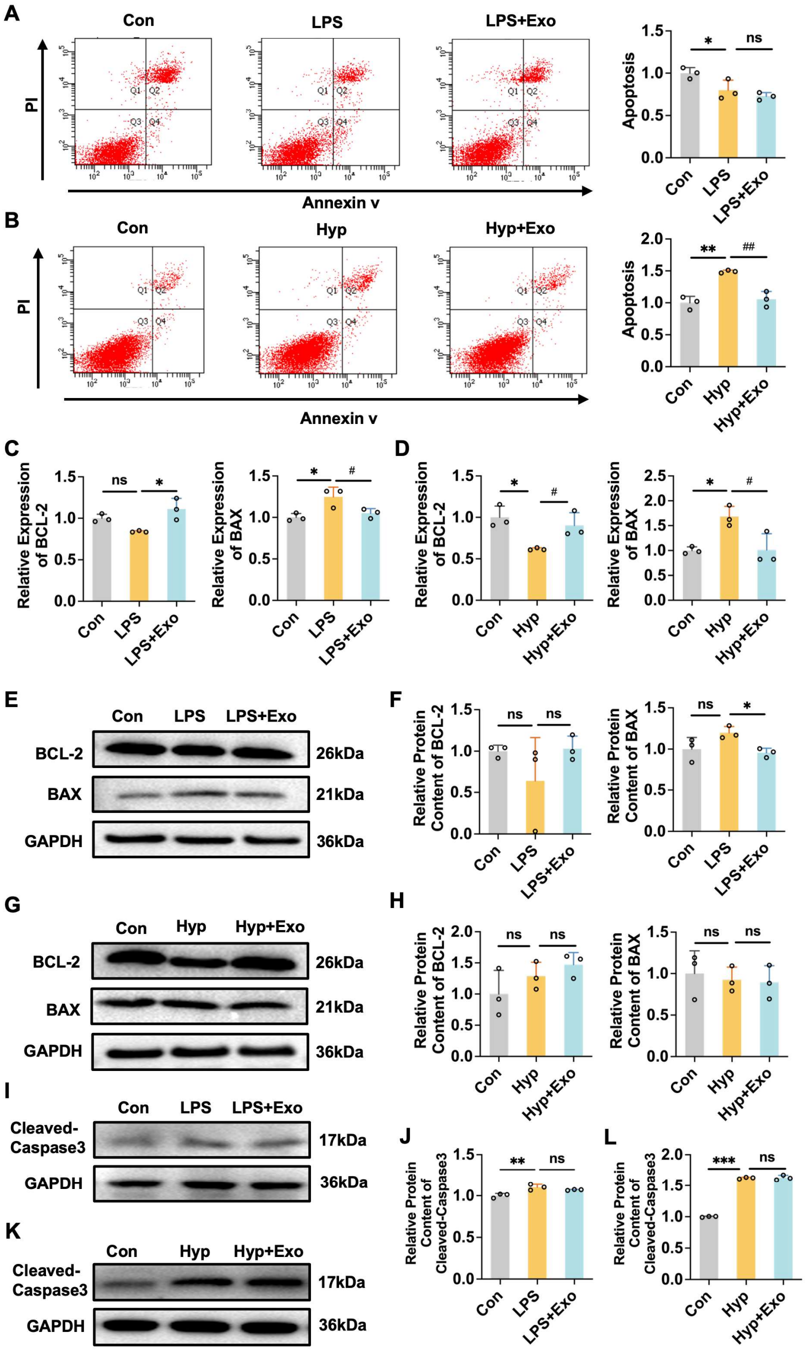

Figure S1. The role of M2-Exo inhibits apoptosis in LPS or hyperoxia induced BPD cell model.

(**A,B**) The apoptosis of MLE-12 cells was detected using flow cytometry (n=3). (**C,D**) BCL-2 and BAX mRNA relative expression (n=3). (**E,G**) Western blots of BCL-2 and BAX (n=3). (**F,H**) Quantification of BCL-2 and BAX protein contents normalized with GAPDH contents. (**I,K**) Western blots of cleaved-caspase3 (n=3). (**J,L**) Quantification of Caspase-3 protein contents normalized with GAPDH contents. One-way ANOVA, ns $p > 0.05$, */# $p < 0.05$, **/## $p < 0.01$, ***$p < 0.001$.

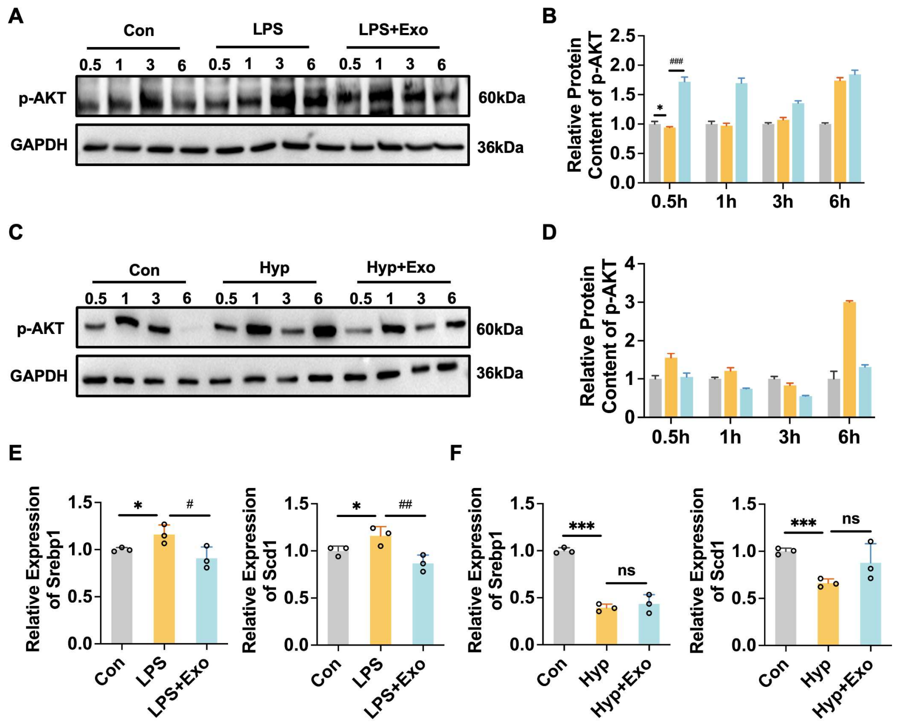

Figure S2. M2-Exo intervention has no role in mTOR signalling pathway.
(**A**,**C**) Western blots of p-AKT at different time points (n=3). (**B**,**D**) Quantifi-cation of p-AKT protein contents normalized with GAPDH contents. (**E**,**F**) Srebp1 and Scd1 mRNA relative expression(n=3). One-way ANOVA, ns $p > 0.05$, */# $p < 0.05$, ## $p < 0.01$, ***/### $p <$ 0.001.
